# Supplementary material for: CRISPR screens reveal genetic determinants of PARP inhibitor sensitivity and resistance in prostate cancer
Source: Nat Commun. 2023 Jan 17;14:252. doi: 10.1038/s41467-023-35880-y (PMC9845315; doi:10.1038/s41467-023-35880-y)

SUPPLEMENTARY INFORMATION

CRISPR screens reveal genetic determinants of PARP inhibitor sensitivity and resistance in prostate cancer

Takuya Tsujino, Tomoaki Takai, Kunihiko Hinohara, Fu Gui, Takeshi Tsutsumi, Xiao Bai, Chenkui Miao, Chao Feng, Bin Gui, Zsofia Sztupinszki, Antoine Simoneau, Ning Xie, Ladan Fazli, Xuesen Dong, Haruhito Azuma, Atish D. Choudhury, Kent W. Mouw, Zoltan Szallasi, Lee Zou, Adam S. Kibel, Li Jia

Supplementary Figures

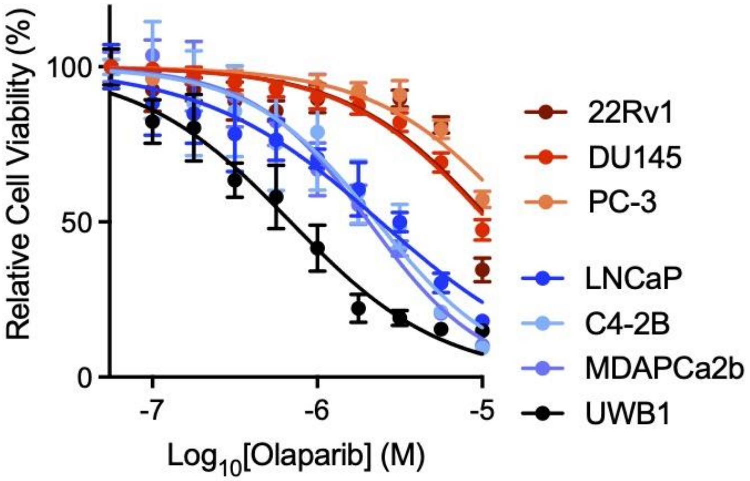

Supplementary Fig. 1 The response of human cancer cell lines to olaparib.
Dose-response curves after treatment with olaparib as indicated for 7 days. Six human prostate cancer (PCa) cell lines (LNCaP, C4-2B, MDAPCa2b, 22Rv1 DU145 and PC-3) and one BRCA1-null human ovarian cancer cell line UWB1.289 (UWB1) were used. Data are presented as mean ± SD (n = 3 biologically independent experiments).

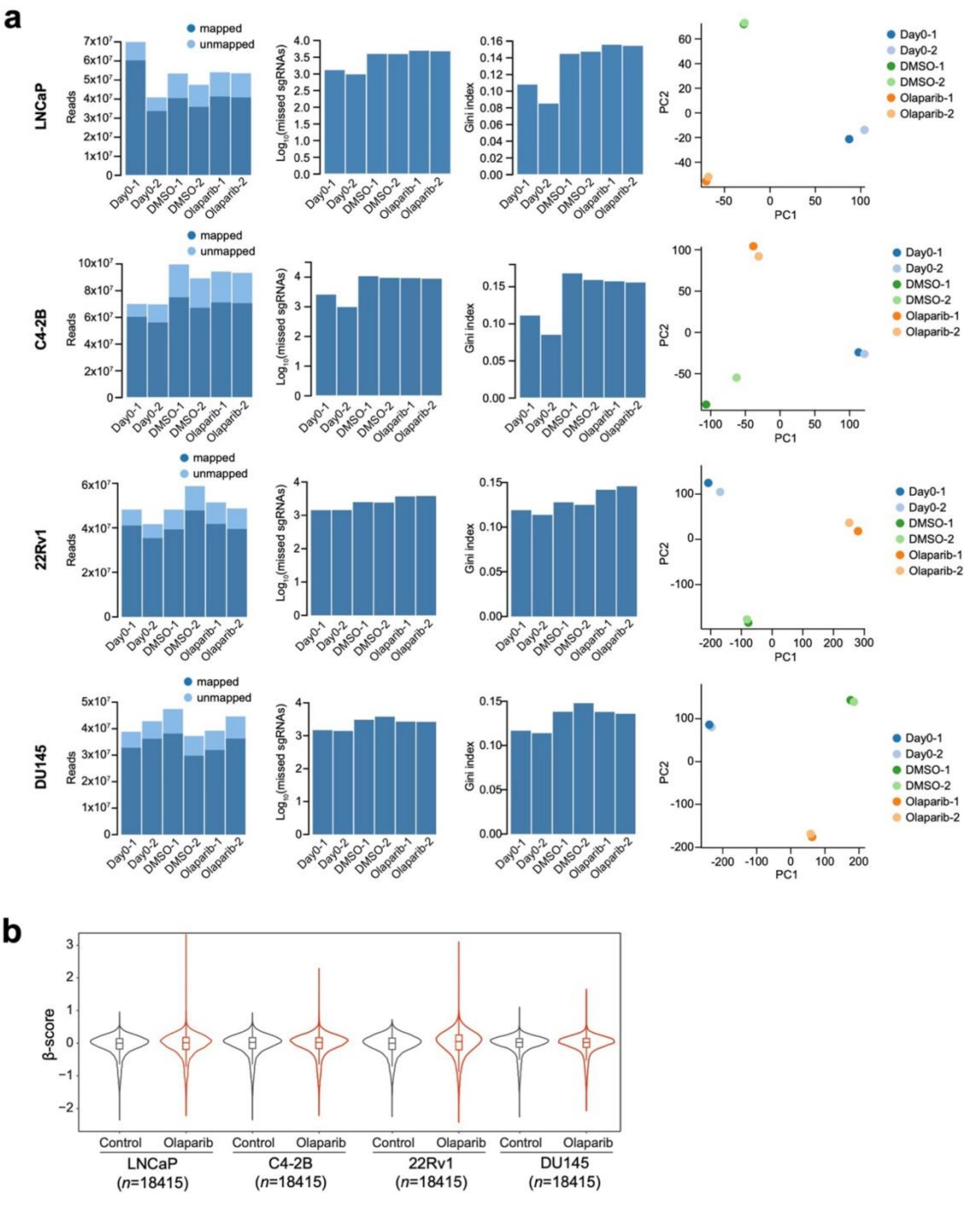

Supplementary Fig. 2 The quality control measurements of CRISPR screens in four PCa cell lines.

a, Read counts of mapped and unmapped sgRNAs, number of missing sgRNAs, Gini index which is the measurement of read evenness within samples, and principal component analysis in each cell line are shown.

b, Violin plots indicating the distribution of β-scores in each screen. Solid lines and boxes inside violin indicate median and interquartile range (IQR), respectively. Whiskers inside violin indicate the range of 1.5 x IQR. N = 18415 targeted genes in the CRISPR library.

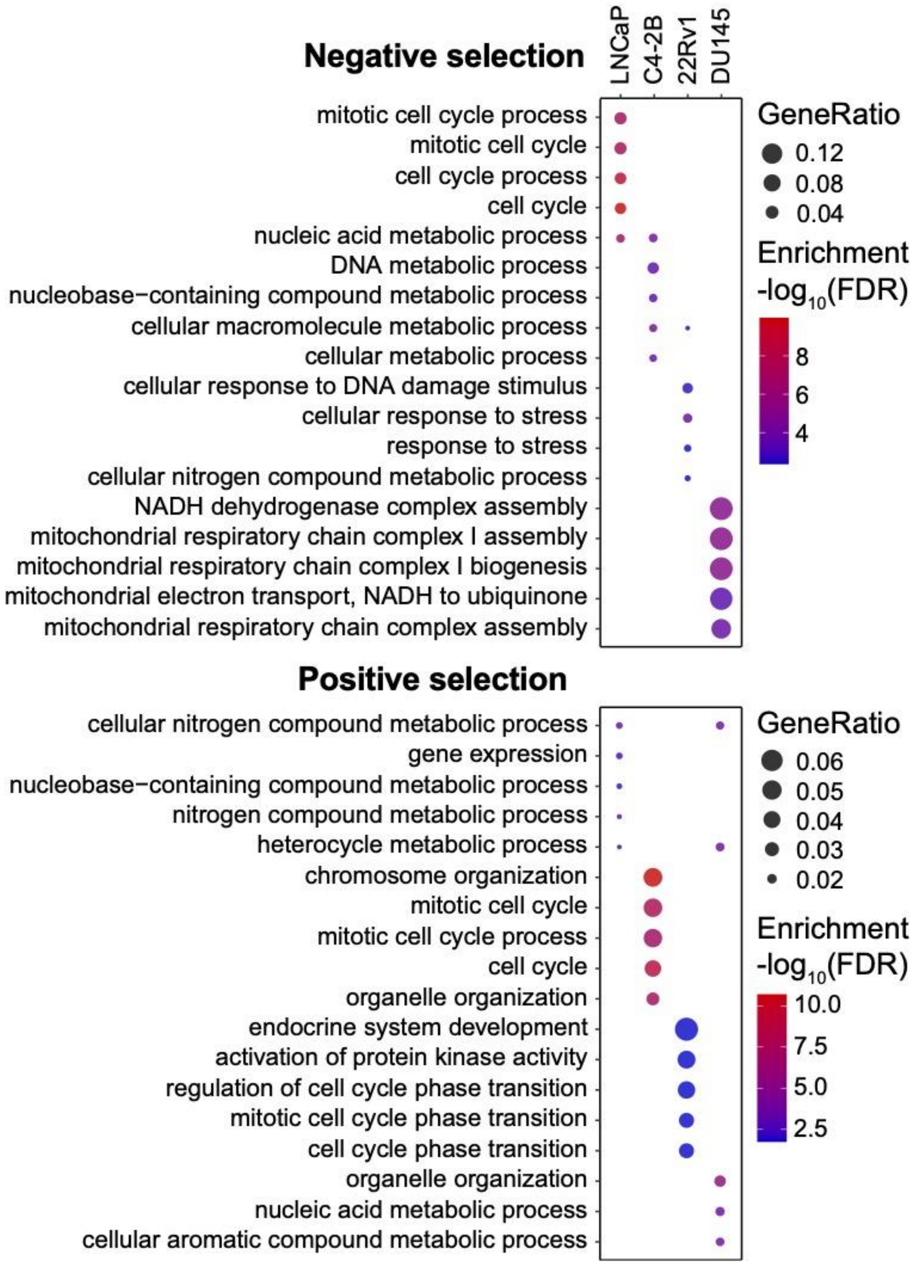

Supplementary Fig. 3 Gene Ontology (GO) analysis of unique genes identified in each CRISPR screen after exclusion of common hits.

Top GO terms enriched in unique negatively selected genes in each cell line after exclusion of common hits (upper panel: LNCaP, n = 167; C4-2B, n = 195; 22Rv1, n = 129; DU145, n = 180). Top GO terms enriched in unique positively selected genes in each cell line after exclusion of common hits (lower panel: LNCaP, n = 211; C4-2B, n = 192; 22Rv1, n = 272; DU145, n = 236). N represents number of identified genes in each cell line.

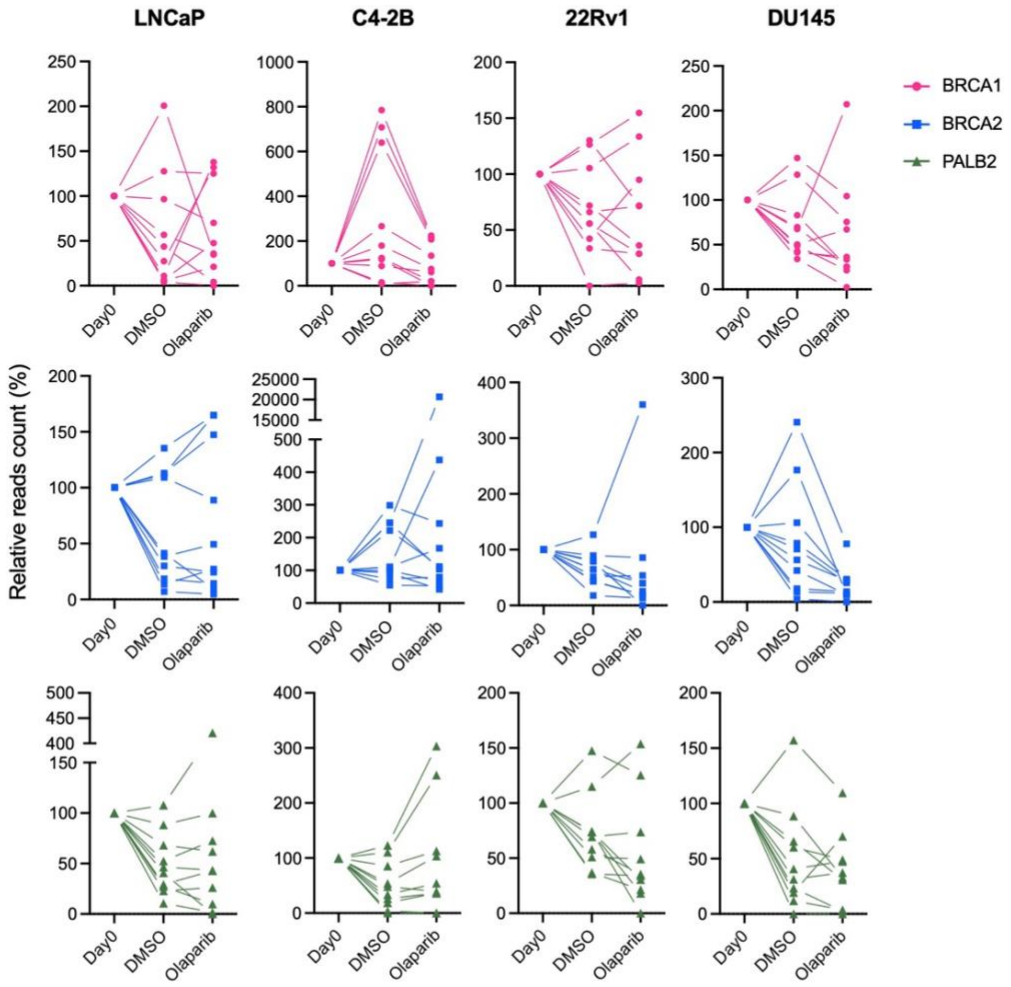

Supplementary Fig. 4 BRCA1, BRCA2, and PALB2 function as fitness genes.

Reads counts of BRCA1, BRCA2, and PALB2 sgRNAs from CRISPR screen samples on day 0 and day 28 with DMSO or olaparib treatment in four cell lines (LNCaP, C4-2B, 22Rv1, and DU145). The relative read count on day 0 is defined as 100.

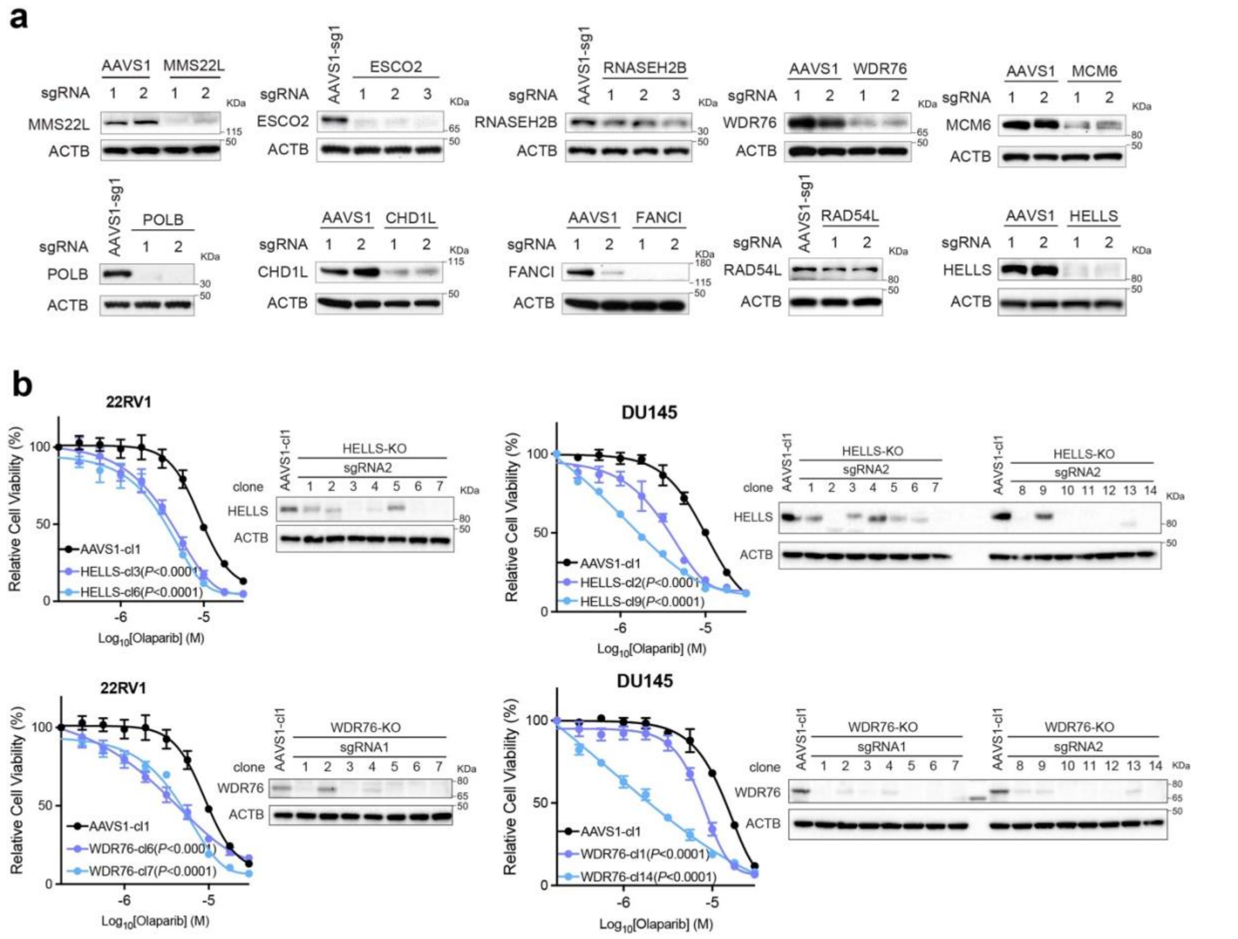

Supplementary Fig. 5 Validation of gene knockout (KO).

a, Immunoblot analyses of the protein level of each gene as indicated after CRISPR/Cas9 KO in C4-2B cells. ACTB (β-actin) is a loading control. At least two sgRNAs were used for each gene.

b, Dose-response curves after treatment with olaparib for AAVS1 control, HELLS-KO, and WDR76-KO 22Rv1 and DU145 cell clones. Immunoblot analysis of the HELLS and WDR76 protein levels in AAVS1 control, HELLS-KO, and WDR76-KO cell clones are shown. Two KO cell clones (cl) were selected for each cell viability experiment as indicated. Data are presented as mean $\pm$ SD (n = 3 biologically independent experiments). The *p*-values were determined by comparing two gene-specific KO cell clones to a control AAVS1 cell clone using two-way ANOVA.

C4-2B MMS22L-KO gating example

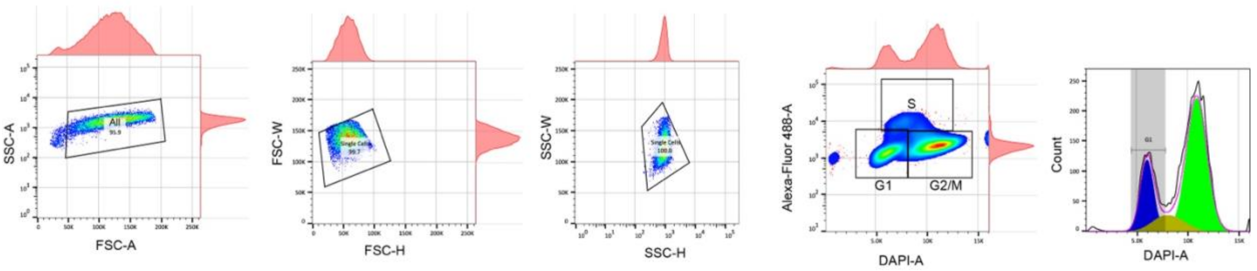

Supplementary Fig. 6 Flow cytometry gating strategy.

Gating approach and representative image of cell cycle analysis by flow cytometry are shown. Prostate cancer cells were labelled using Click-iT™ EdU Flow Cytometry Kit. Population of each cell cycle phase was analyzed using FlowJo Software (Vestion10.7.1).

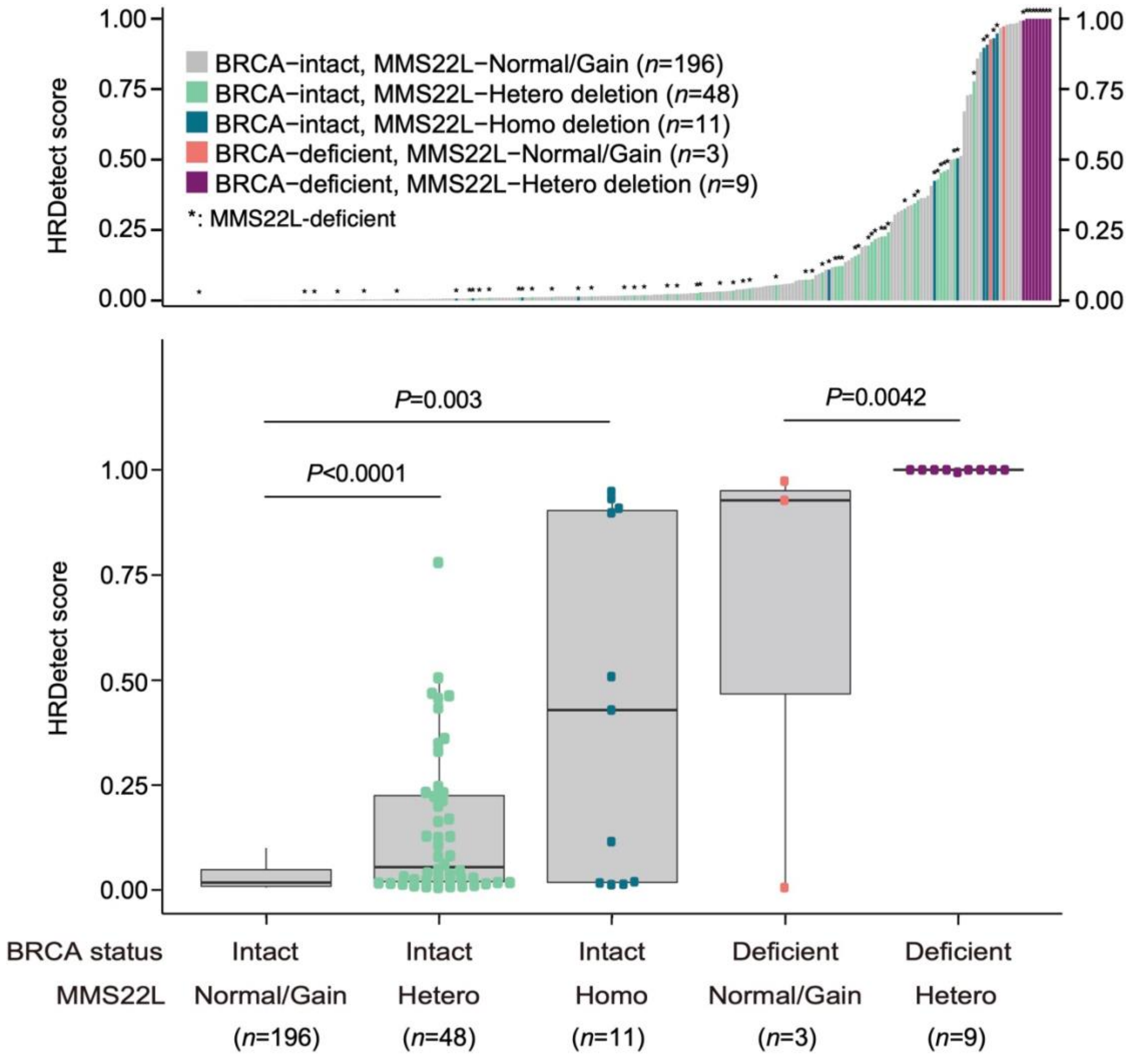

Supplementary Fig. 7 HRDetect scores in prostate tumors with BRCA and/or MMS22L alterations.

Ranked HRDetect scores (upper panel) in prostate tumors with BRCA and/or MMS22L genomic alterations as indicated. Comparison of HRDetect scores (lower panel) between five patient groups classified based BRCA and MMS22L status (n = 196, 48, 11, 3, and 9 tumor samples in each group, respectively). HRDetect score is calculated using whole-genome sequencing data with HRDetect algorithm [1-3]. Data are presented as boxplot indicating median, 25th-75th percentile (box) and minimum and maximum values (whiskers). The *p*-values were determined using unpaired two-sided *t*-test.

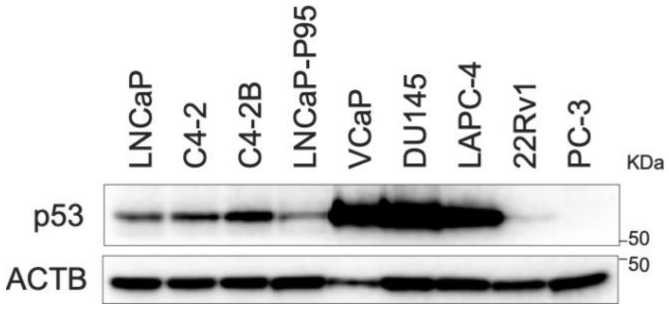

Supplementary Fig. 8 p53 protein expression in PCa cell lines.

Immunoblot analysis of endogenous p53 protein expression in PCa cell lines as indicated. ACTB (β-actin) is a loading control.

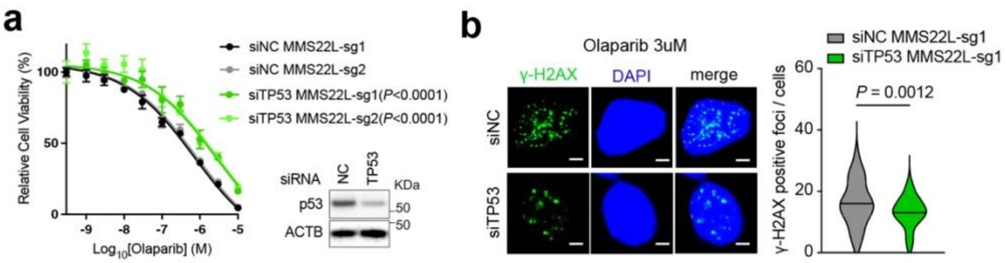

Supplementary Fig. 9 Knockdown of TP53 expression reduces olaparib sensitivity and increases DNA damage in MMS22L-KO cells.

a, Dose-response curves after treatment with olaparib as indicated for 7 days in MMS22L-KO C4-2B cells transfected with siRNA against TP53 or negative control (NC). Two MMS22L-KO cell lines (sg1 and sg2) were used. The *p*-values were determined by comparing siTP53 to siNC in each MMS22L-KO cell line using two-way ANOVA. Data are presented as mean ± SD (n = 3 biologically independent experiments). Immunoblot analysis showing p53 knockdown efficiency.
b, Representative images (left panel) and quantification (right panel) of γ-H2AX foci in MMS22L-KO C4-2B cells transfected with siTP53 or siNC after treatment with olaparib (3 μM) for 24 h. More than 100 cells were analyzed per condition. Solid lines inside the violin indicate the median. The experiment was repeated independently twice with similar results. Scale bar = 5 μm.

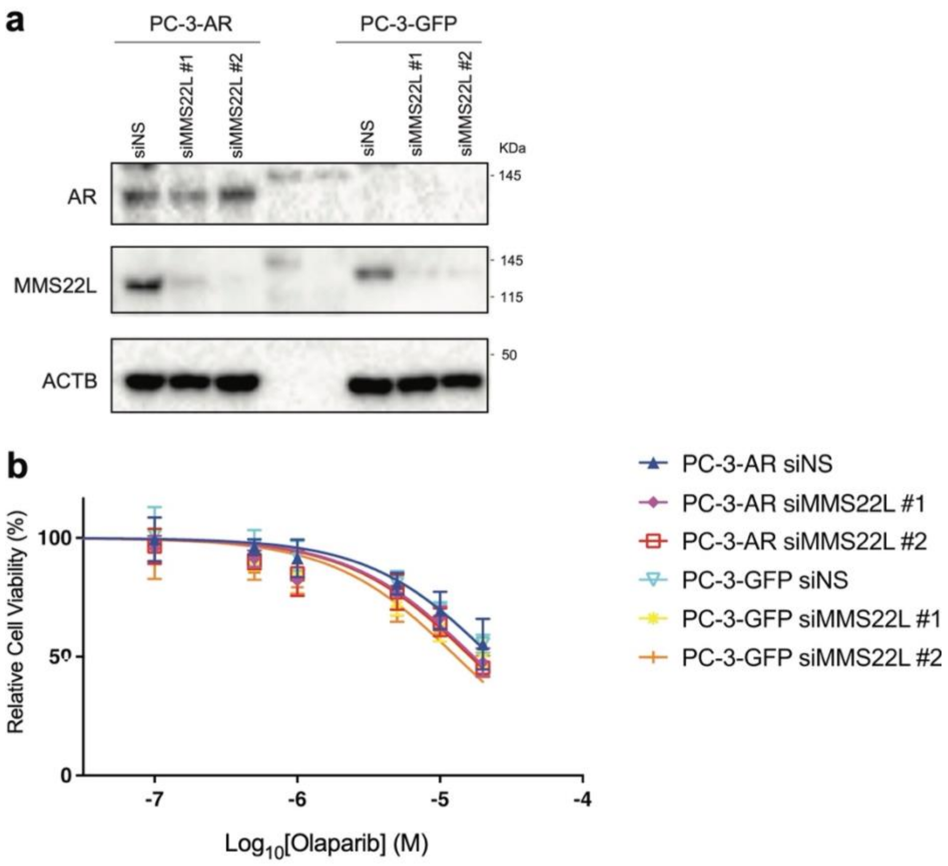

Supplementary Fig. 10 AR restoration does not impact olaparib response in AR-negative PC-3 cells.

a, AR-negative (PC-3-GFP) and AR-expressing (PC-3-AR) PC-3 cell lines were generated by stably infected viruses containing GFP or AR gene. The AR expression level was determined by immunoblot in both cell lines. MMS22L knockdown was confirmed in cells transfected with siRNA against MMS22L (#1 and #2) or negative control (NC) for 2 days.

b, Dose-response curves after treatment with olaparib as indicated for 5 days in PC-3-GFP and PC-3-AR cells transfected with siMMS22L or siNC. Data are presented as mean ± SD (n = 3 biologically independent experiments).

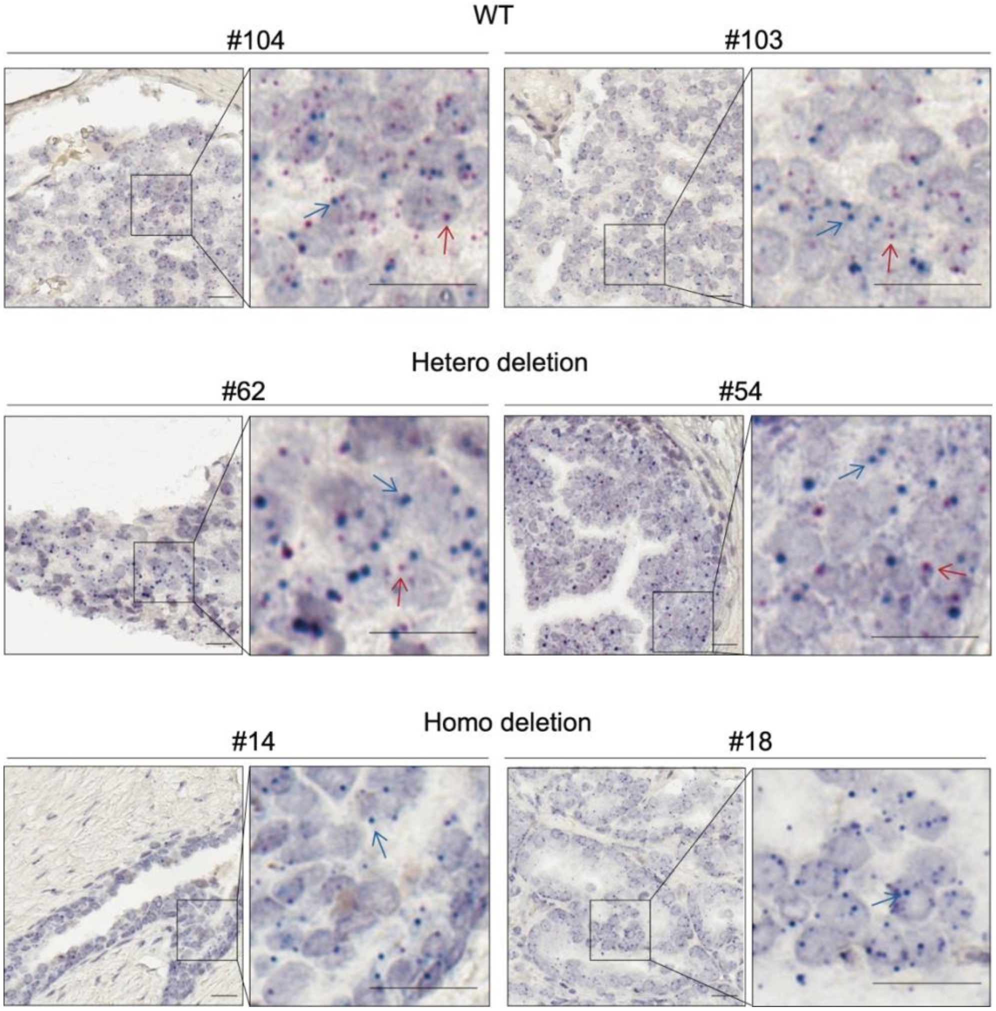

Supplementary Fig. 11 DNAscope in situ hybridazation on PCa tissue microarray (TMA). Six representative MMS22L DNAscope images from MMS22L wild-type (WT, #104 & #103), heterozygous deletion (Hetero, #62 & #54), and homozygous deletion (Homo, #14 & #18) cases are presented. Red signals (red arrow) indicate a probe targeting the MMS22L gene on chromosome 6q. Blue signals (blue arrow) indicate a chromosome enumeration control probe targeting the centromeric region of chromosome 6p (CEP6p). The number of red and blue dots were counted, and the ratio of red/blue was calculated. WT, red/blue $\geq$ 0.5; Hetero deletion, red/blue between 0.1-0.5; Homo deletion, red/blue $\leq$ 0.1. Scale bar = 20μm.

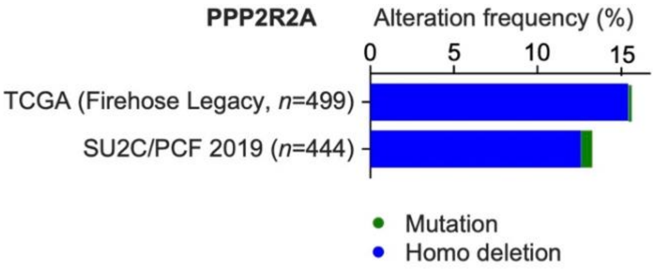

Supplementary Fig. 12 PPP2R2A genomic alterations in PCa.

Frequency of PPP2R2A mutation and homozygous (Homo) deletion in the TCGA (Firehose Legacy) and SU2C/PCF cohorts [4,5].

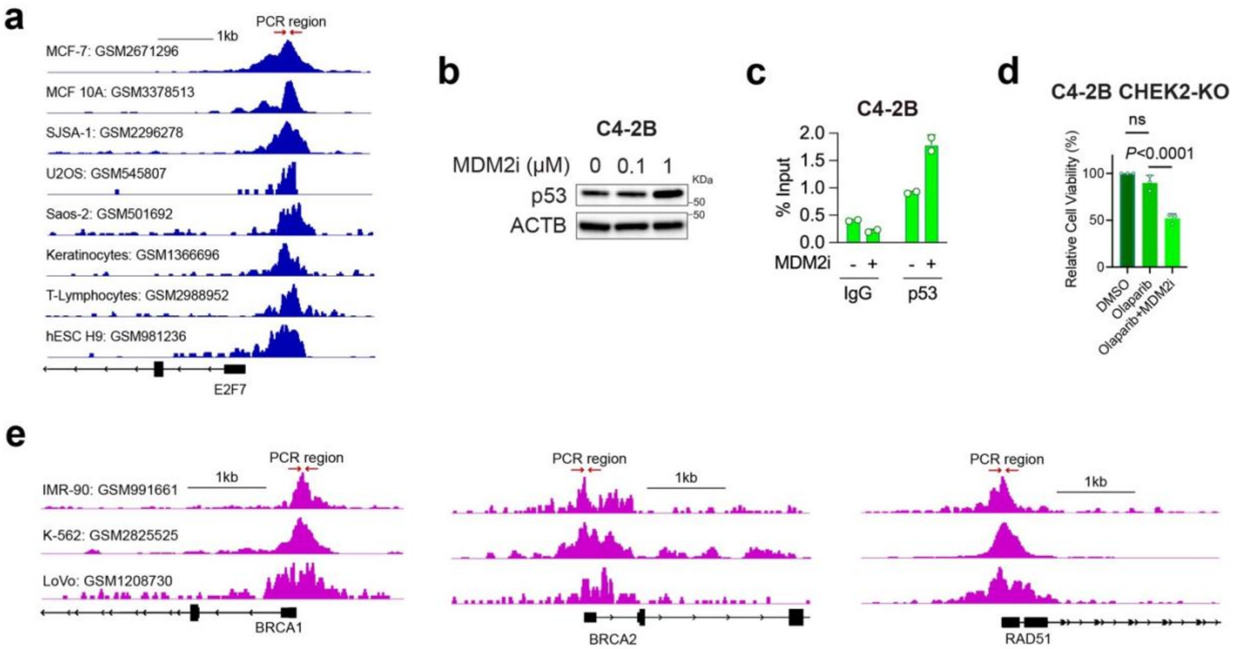

Supplementary Fig. 13 p53- and E2F7-mediated gene regulation in PCa cells.

a, Genome browser view of p53 ChIP-seq signals at the E2F7 promoter in the indicated cell lines. The ChIP-seq data were obtained from publicly available datasets as indicated [6-13]. All data were obtained from ChIP-Atlas (http://chip-atlas.org) [14]. Red arrows indicate the genomic region used for ChIP-qPCR validation in **Fig. 7d**.

b, Immunoblot analysis of p53 in C4-2B cells after treatment with MDM2 inhibitor (MDM2i) nutlin as indicated.

c, p53 ChIP-qPCR was performed at the E2F7 promoter region in C4-2B cells after treatment with nutlin (1 μM) or vehicle for 24 h. p53 occupancy was significantly increased at the E2F7 promoter after MDM2 inhibition. Data are presented as mean ± SD of two independent experiments.

d, Cell viability after treatment with olaparib (5 μM) in the presence or absence of nutlin (1 μM) in CHEK2-KO C4-2B cells. Data are presented as mean ± SD (n = 3 biologically independent experiments). The *p*-value was determined using unpaired two-sided *t*-test.

e, Genome browser view of E2F7 ChIP-seq signals at the promote regions of BRCA1, BRCA2, and RAD51 genes in the indicated cell lines. The ChIP-seq data were obtained from publicly available datasets as indicated [15-17]. All data were obtained from ChIP-Atlas (http://chip-atlas.org) [14]. Red arrows indicate the genomic region used for ChIP-qPCR validation in **Fig. 7f**.

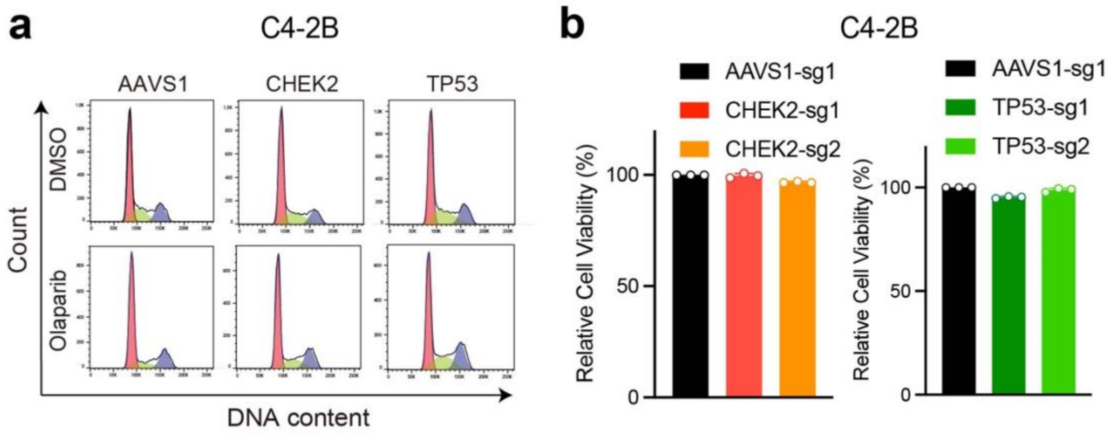

Supplementary Fig. 14 Loss of CHEK2/TP53 has no effect on PCa cell cycle and growth.

a, Cell cycle analysis in AAVS1 control, CHEK2-KO, and TP53-KO C4-2B cells after treatment with DMSO or olaparib (5 μM) for 72 hours**.**

b, Cell viability of AAVS1 control, CHEK2-KO, and TP53-KO C4-2B cells under regular cell culture condition for 5 days. Data are presented as mean ± SD (n = 3 biologically independent experiments).

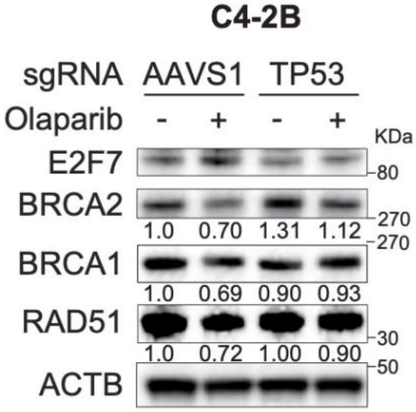

Supplementary Fig. 15 Loss of TP53 increases HRR gene expression.
Immunoblot analysis of the indicated proteins in AAVS1 control and TP53-KO C4-2B cells after treatment with olaparib (1 μM) or vehicle for 72h. The protein levels of BRCA1, BRCA2, and RAD51 are decreased after olaparib treatment in TP53-intact AAVS1 control cells but remained at a high level in TP53-KO cells. The integrated optical density (IOD) values of the indicated proteins normalized by ACTB IOD are shown.

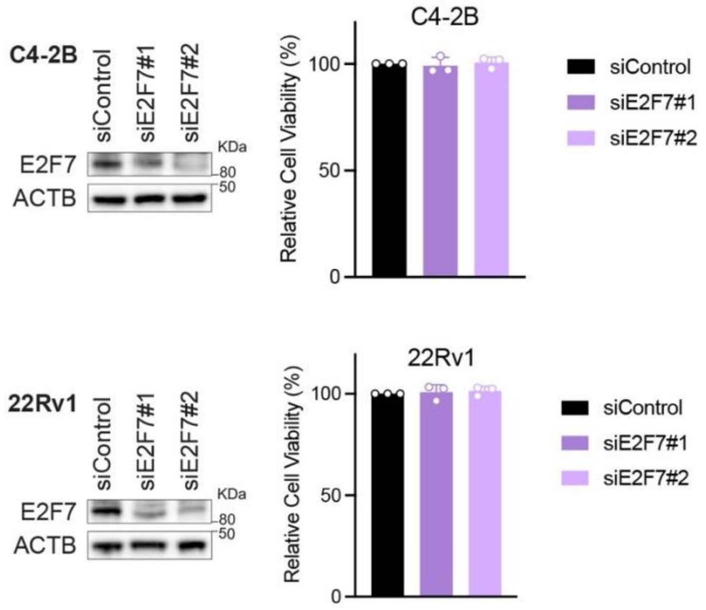

Supplementary Fig. 16 Knockdown of E2F7 has no effect on PCa cell growth. Immunoblot analyses (left panel) of the E2F7 protein levels in C4-2B and 22Rv1 cells after transfection with siRNAs against E2F7 (#1 or #2) or negative control for 2 days. Cell viability (right panel) of C4-2B and 22Rv1 cells after transfection with siRNAs as indicated for 5 days. Data are presented as mean ± SD (n = 3 biologically independent experiments).

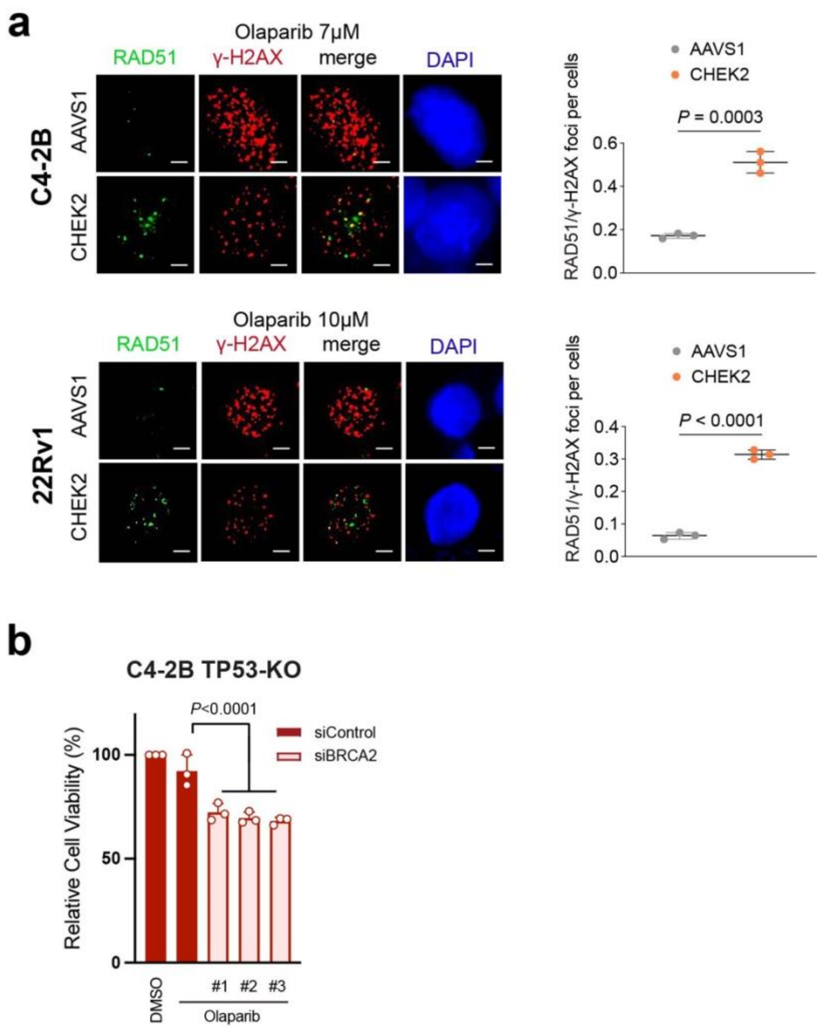

Supplementary Fig. 17 Loss of CHEK2 enhances HRR capacity.

a. Representative images (left panel) showing double immunofluorescence staining of concurrent RAD51 (green) and γ-H2AX (red) foci in AAVS1 control and CHEK2-KO C4-2B and 22Rv1 cells after treatment with olaparib (7 μM for C4-2B and 10 μM for 22Rv1) for 24 h. Quantification of the ratio of RAD51/γ-H2AX foci (right panel). Dots indicate each replicate with more than 100 cells analyzed. Data are presented as mean ± SD of three biologically independent experiments. The *p*-values were determined using unpaired two-sided *t*-test. Scale bar = 5 μm.

b, Cell viability of TP53-KO C4-2B cells transfected with siRNAs (#1, #2, or #3) against BRCA2 or negative control. Cells were treated with olaparib (1 μM) for 7 days. Data are presented as mean ± SD (n = 3 biologically independent experiments). The *p*-value was determined using unpaired two-sided *t*-test.

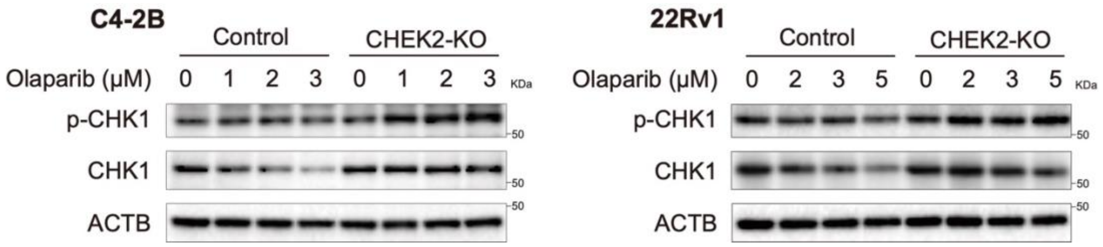

Supplementary Fig. 18 Olaparib treatment induces ATR activity.
Immunoblot analyses of p-CHK1 and total CHK1 in AAVS1 control and CHEK2-KO C4-2B and 22Rv1 cells after olaparib treatment as indicated.

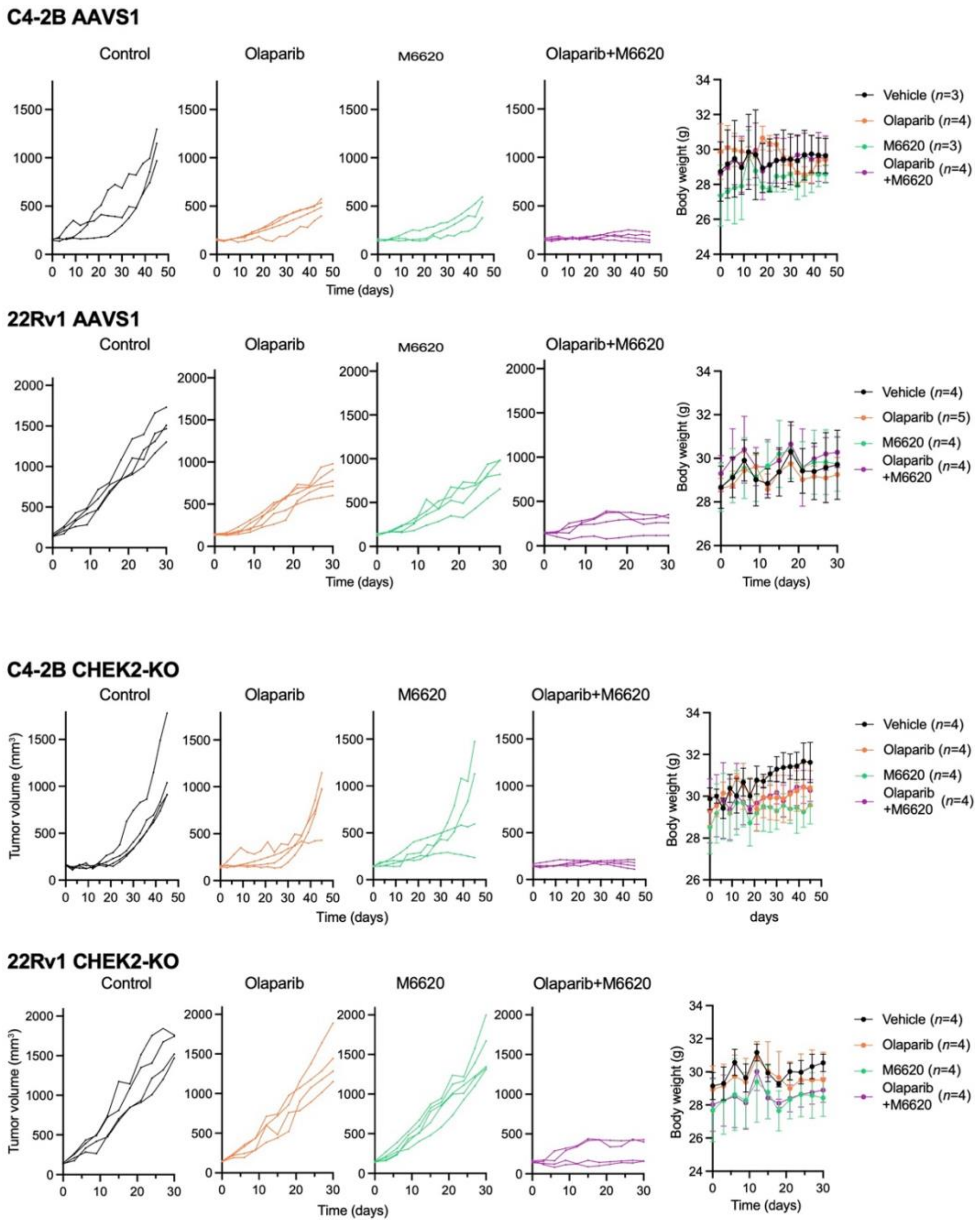

Supplementary Fig. 19 Combination treatment with olaparib and M6620 in AAVS1 control and CHEK2-KO xenograft models.

Tumor growth of AAVS1 control and CHEK2-KO C4-2B and 22Rv1 tumors in each mouse treated with the indicated agents (n = 3, 4, 3, 4 in each group for C4-2B AAVS1; n = 4, 5, 4, 4 in each group for 22Rv1 AAVS1; n = 4, 4, 4, 4 in each group for C4-2B CHEK2-KO; n = 4, 4, 4, 4

in each group for 22Rv1 CHEK2-KO). Mean body weight of the mice are shown. Body weight is shown as mean ± SD of each time point.

References

1 Sztupinszki, Z. *et al.* Detection of Molecular Signatures of Homologous Recombination Deficiency in Prostate Cancer with or without BRCA1/2 Mutations. *Clin Cancer Res* **26**, 2673-2680, doi:10.1158/1078-0432.CCR-19-2135 (2020).

2 Sztupinszki, Z. *et al.* Migrating the SNP array-based homologous recombination deficiency measures to next generation sequencing data of breast cancer. *NPJ Breast Cancer* **4**, 16, doi:10.1038/s41523-018-0066-6 (2018).

3 Davies, H. *et al.* HRDetect is a predictor of BRCA1 and BRCA2 deficiency based on mutational signatures. *Nat Med* **23**, 517-525, doi:10.1038/nm.4292 (2017).

4 Hoadley, K. A. *et al.* Cell-of-Origin Patterns Dominate the Molecular Classification of 10,000 Tumors from 33 Types of Cancer. *Cell* **173**, 291-304 e296, doi:10.1016/j.cell.2018.03.022 (2018).

5 Abida, W. *et al.* Genomic correlates of clinical outcome in advanced prostate cancer. *Proc Natl Acad Sci U S A* **116**, 11428-11436, doi:10.1073/pnas.1902651116 (2019).

6 Hafner, A. *et al.* p53 pulses lead to distinct patterns of gene expression albeit similar DNA-binding dynamics. *Nat Struct Mol Biol* **24**, 840-847, doi:10.1038/nsmb.3452 (2017).

7 Karsli Uzunbas, G., Ahmed, F. & Sammons, M. A. Control of p53-dependent transcription and enhancer activity by the p53 family member p63. *J Biol Chem* **294**, 10720-10736, doi:10.1074/jbc.RA119.007965 (2019).

8 Andrysik, Z. *et al.* Identification of a core TP53 transcriptional program with highly distributed tumor suppressive activity. *Genome Res* **27**, 1645-1657, doi:10.1101/gr.220533.117 (2017).

9 Smeenk, L. *et al.* Role of p53 serine 46 in p53 target gene regulation. *PLoS One* **6**, e17574, doi:10.1371/journal.pone.0017574 (2011).

10 Koeppel, M. *et al.* Crosstalk between c-Jun and TAp73alpha/beta contributes to the apoptosis-survival balance. *Nucleic Acids Res* **39**, 6069-6085, doi:10.1093/nar/gkr028 (2011).

11 McDade, S. S. *et al.* Genome-wide characterization reveals complex interplay between TP53 and TP63 in response to genotoxic stress. *Nucleic Acids Res* **42**, 6270-6285, doi:10.1093/nar/gku299 (2014).

12 Nguyen, T. T. *et al.* Revealing a human p53 universe. *Nucleic Acids Res* **46**, 8153-8167, doi:10.1093/nar/gky720 (2018).

13 Akdemir, K. C. *et al.* Genome-wide profiling reveals stimulus-specific functions of p53 during differentiation and DNA damage of human embryonic stem cells. *Nucleic Acids Res* **42**, 205-223, doi:10.1093/nar/gkt866 (2014).

14 Oki, S. *et al.* ChIP-Atlas: a data-mining suite powered by full integration of public ChIP-seq data. *EMBO Rep* **19**, doi:10.15252/embr.201846255 (2018).

15 Aksoy, O. *et al.* The atypical E2F family member E2F7 couples the p53 and RB pathways during cellular senescence. *Genes Dev* **26**, 1546-1557, doi:10.1101/gad.196238.112 (2012).

16 Consortium, E. P. An integrated encyclopedia of DNA elements in the human genome. *Nature* **489**, 57-74, doi:10.1038/nature11247 (2012).

17 Yan, J. *et al.* Transcription factor binding in human cells occurs in dense clusters formed around cohesin anchor sites. *Cell* **154**, 801-813, doi:10.1016/j.cell.2013.07.034 (2013).

SUPPLEMENTARY SOURCE DATA

Supplementary Fig. 5a

Uncropped images of all immunoblots. The dotted rectangles are the areas shown in Fig. 5a. Protein expression of each lane is derived from the same protein extraction.

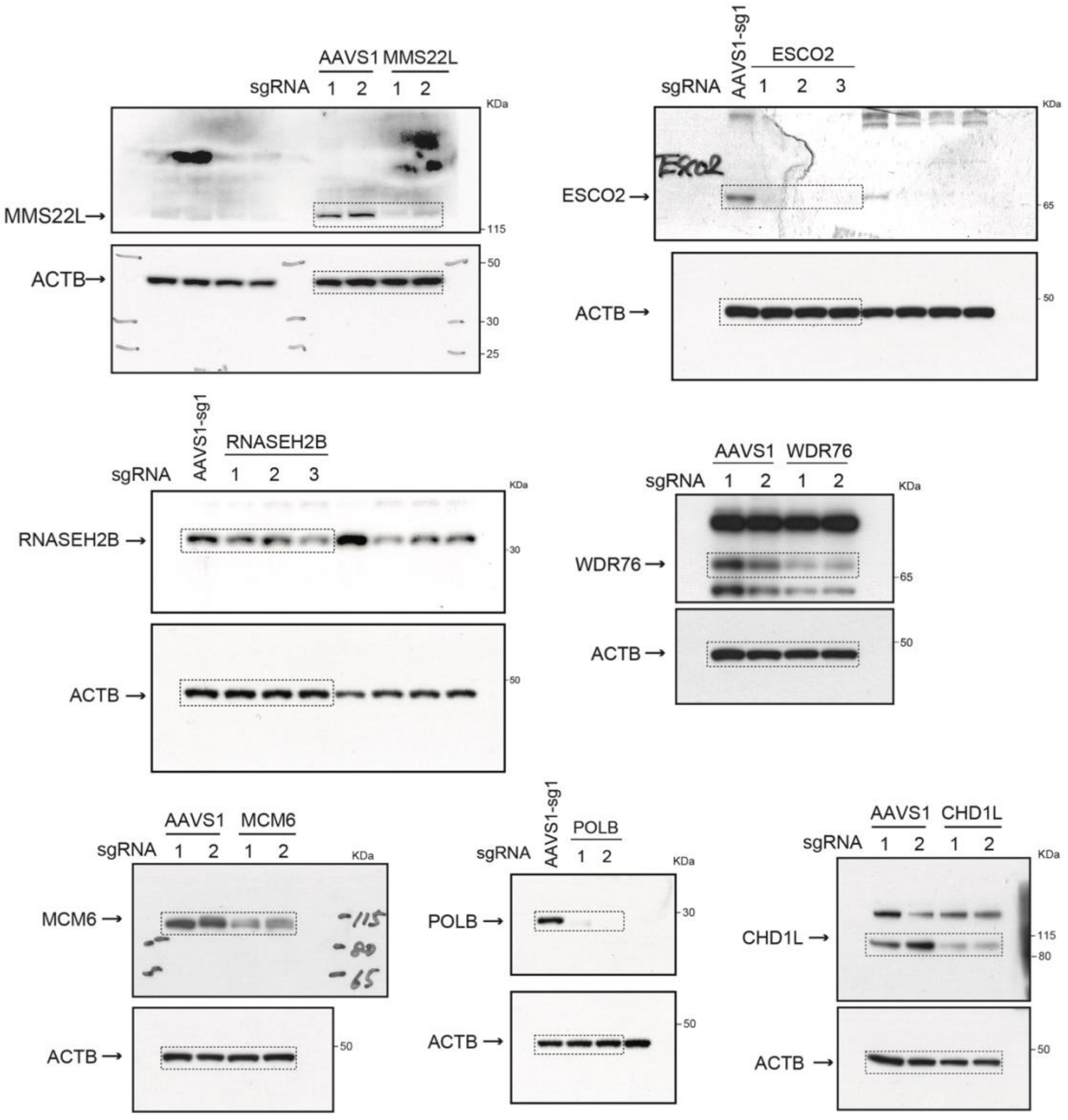

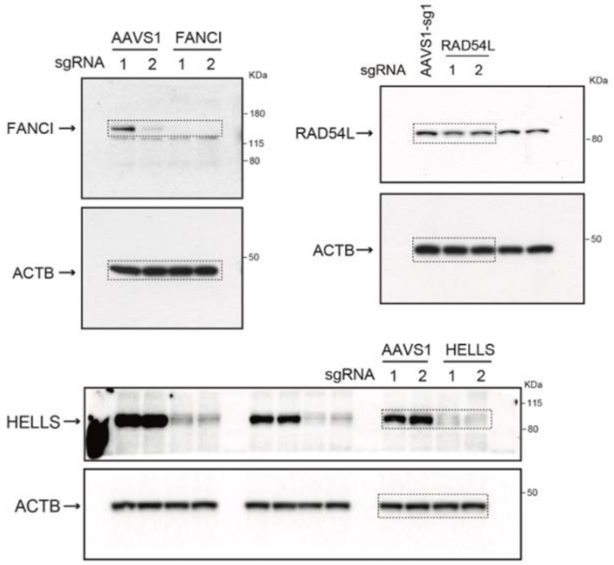
AAVS1
FANCI
sgRNA
1
2
1
2
KDa
180
115
80
FANCI
ACTB
50
AAVS1-sg1
RAD54L
sgRNA
1
2
KDa
RAD54L
80
ACTB
50
AAVS1
HELLS
sgRNA
1
2
1
2
KDa
115
80
HELLS
ACTB
50

Supplementary Fig. 5b

Uncropped images of all immunoblots. The dotted rectangles are the areas shown in Fig. 5b. Protein expression of each lane is derived from the same protein extraction.

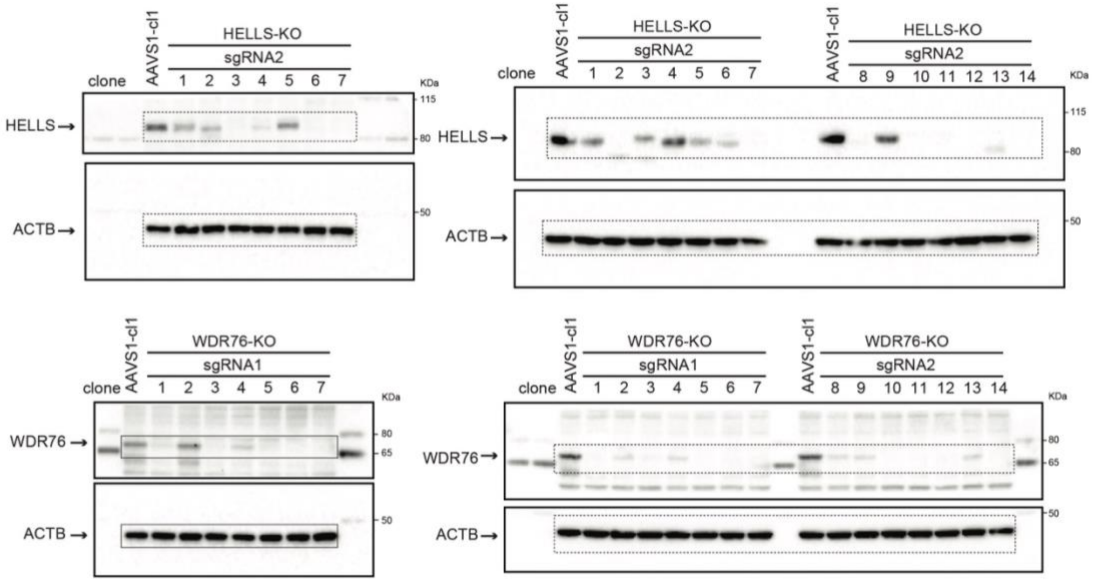

Supplementary Fig. 8

Uncropped images of all immunoblots. The dotted rectangles are the areas shown in Fig. 8. Protein expression of each lane is derived from the same protein extraction.

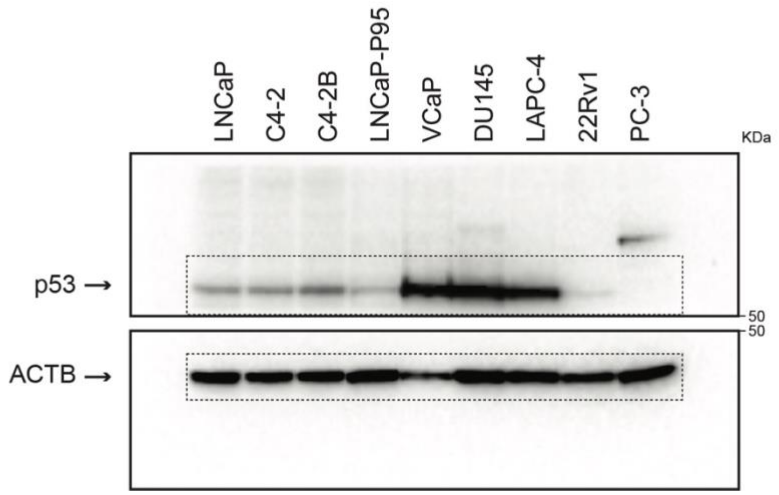

Supplementary Fig. 9

Uncropped images of all immunoblots. The dotted rectangles are the areas shown in Fig. 9. Protein expression of each lane is derived from the same protein extraction.

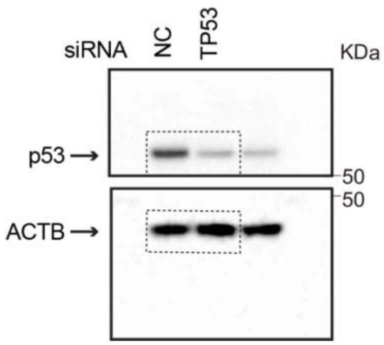

Supplementary Fig. 10

Uncropped images of all immunoblots. The dotted rectangles are the areas shown in Fig. 10. Protein expression of each lane is derived from the same protein extraction.

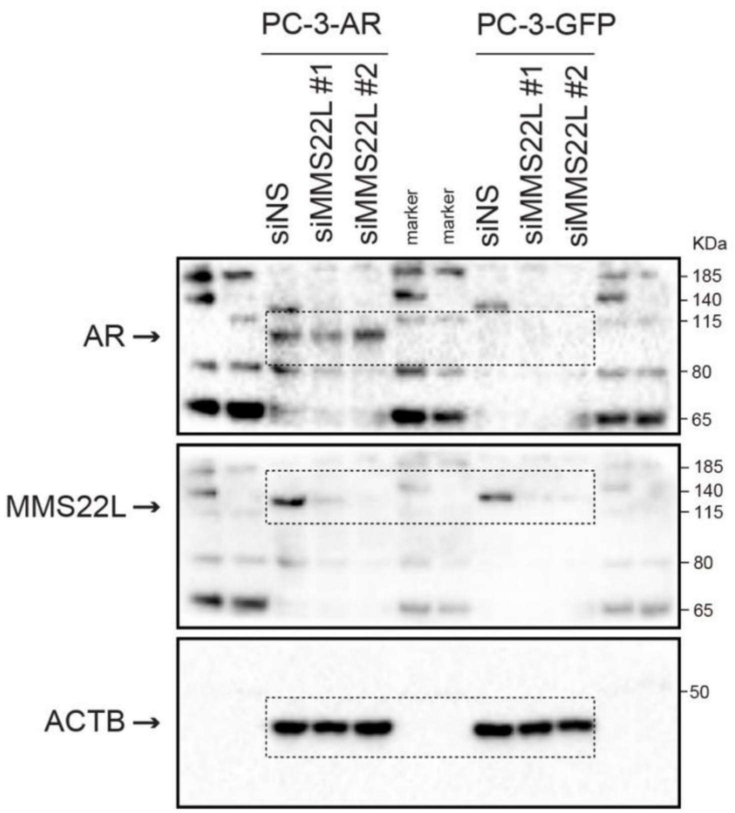

Supplementary Fig. 13

Uncropped images of all immunoblots. The dotted rectangles are the areas shown in Fig. 13. Protein expression of each lane is derived from the same protein extraction.

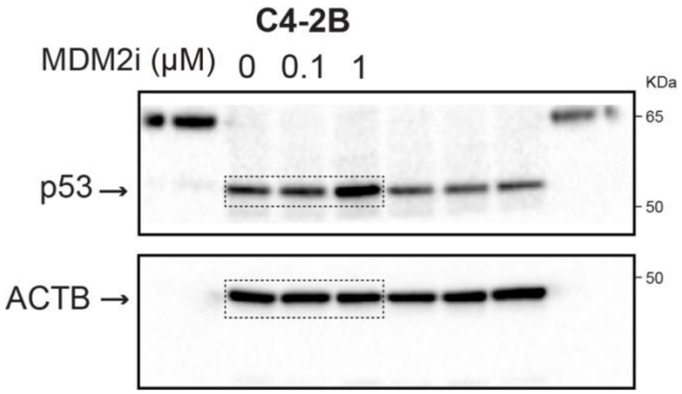

Supplementary Fig. 15

Uncropped images of all immunoblots. The dotted rectangles are the areas shown in Fig. 15. Protein expression of each lane is derived from the same protein extraction. BRCA2, RAD51, and ACTB are run on the same gel. BRCA1 and E2F7 are run on the same gel.

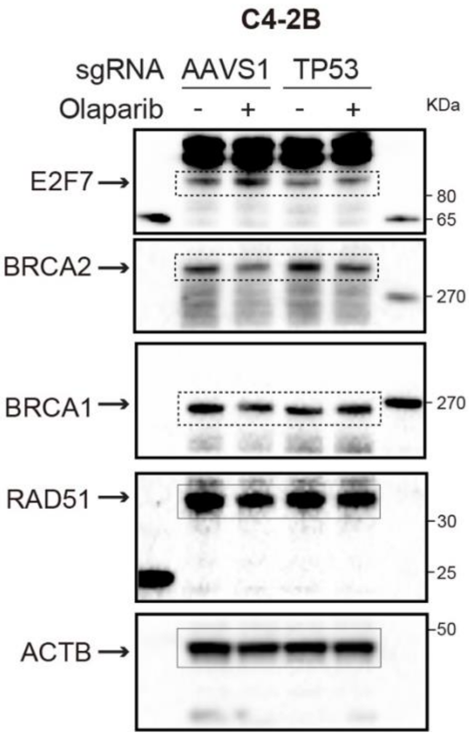

Supplementary Fig. 16

Uncropped images of all immunoblots. The dotted rectangles are the areas shown in Fig. 16. Protein expression of each lane is derived from the same protein extraction.

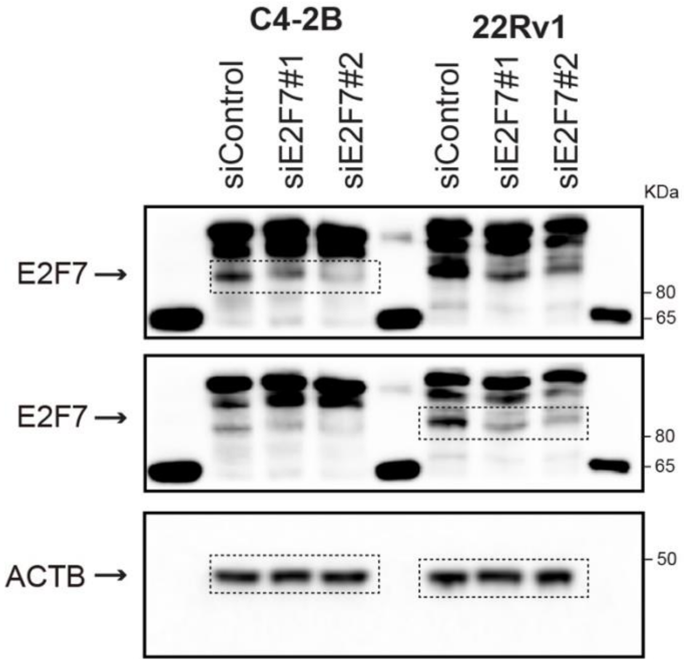

Supplementary Fig. 18

Uncropped images of all immunoblots. The dotted rectangles are the areas shown in Fig. 18. Protein expression of each lane is derived from the same protein extraction.

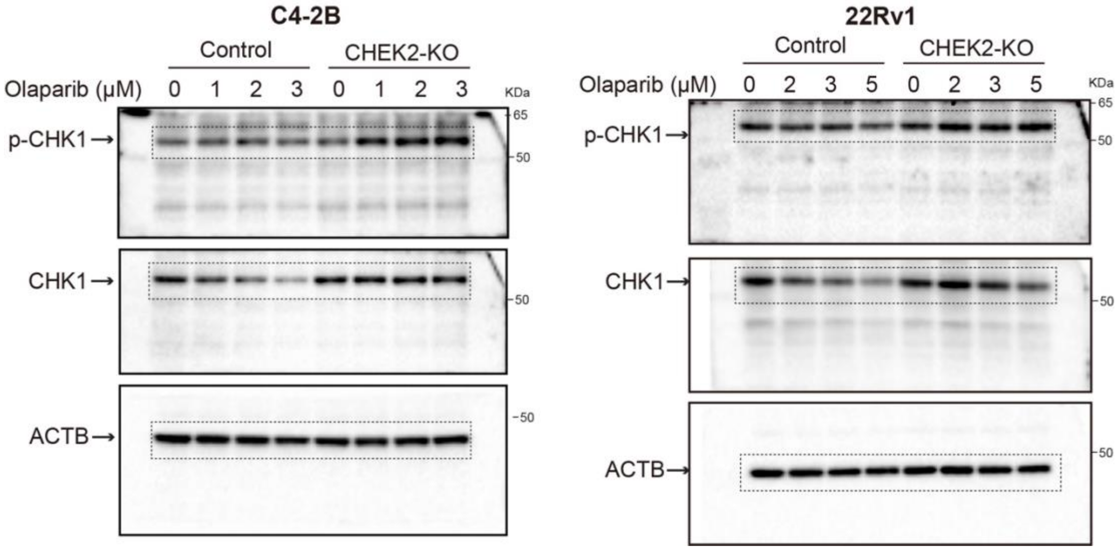

Supplement: Supplementary file 1 — Supplementary Information [file 41467_2023_35880_MOESM1_ESM.pdf]
