# Supplementary material for: CRISPR screens reveal genetic determinants of PARP inhibitor sensitivity and resistance in prostate cancer
Source: Nat Commun. 2023 Jan 17;14:252. doi: 10.1038/s41467-023-35880-y (PMC9845315; doi:10.1038/s41467-023-35880-y)
Supplement: Supplementary file 2 — Description of Additional Supplementary Files [file 41467_2023_35880_MOESM2_ESM.pdf]

Title: Supplementary Data 1:
Description: Genetic alterations of DNA damage response (DDR) genes in prostate cancer cell lines (Exome sequencing data)

Title: Supplementary Data 2:
Description: Genes identified from negative selection (CRISPR screen data)

Title: Supplementary Data 3:
Description: Common negatively selected genes (CRISPR screen data)

Title: Supplementary Data 4:
Description: Genes identified from positive selection (CRISPR screen data)

Title: Supplementary Data 5:
Description: Common positively selected genes (CRISPR screen data)

Title: Supplementary Data 6:
Description: Olaparib upregulated genes (RNA-seq data)

Title: Supplementary Data 7:
Description: Materials (antibodies, small molecule inhibitors, kits, cell lines, siRNA sequences, sgRNA sequences, primers, vectors)
